# Supplementary material for: The Upconversion Luminescence of Ca3Sc2Si3O12:Yb3+,Er3+ and Its Application in Thermometry
Source: Nanomaterials (Basel). 2023 Jun 22;13(13):1910. doi: 10.3390/nano13131910 (PMC10343700; doi:10.3390/nano13131910)
Supplement: Supplementary file 1 [file nanomaterials-13-01910-s001.zip › nanomaterials-2451227-supplementary.pdf]

nanomaterials

Supplementary Materials

The Upconversion Luminescence of $Ca_3Sc_2Si_3O_{12}$:Yb^{3+},Er^{3+} and Its Application in Thermometry

Junyu Hong [1], Feilong Liu [2], Miroslav D. Dramićanin [3], Lei Zhou [1,*] and Mingmei Wu [1]

[1] School of Chemical Engineering and Technology, Sun Yat-sen University, Zhuhai 519082, China; hongjy23@mail.sysu.edu.cn (J.H.); ceswmm@mail.sysu.edu.cn (M.W.)
[2] School of Marine Sciences, Sun Yat-sen University, Zhuhai 519082, China; liuflong3@mail2.sysu.edu.cn
[3] Center of Excellence for Photoconversion, Vinča Institute of Nuclear Sciences-National Institute of the Republic of Serbia, University of Belgrade, PO Box 522, 11,001 Belgrade, Serbia; dramican@vinca.rs
* Correspondence: zhoul8@mail.sysu.edu.cn

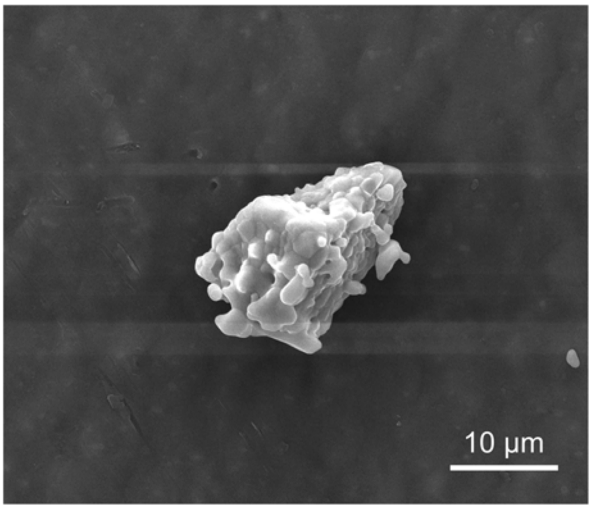

Figure S1. SEM image of CSS:0.2Yb^{3+},0.02Er^{3+}.

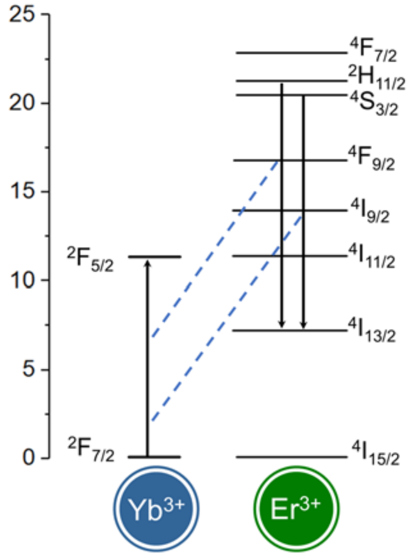

Figure S2. the cross-relaxation of $^2H_{11/2}$/$^4S_{3/2}$ (Er^{3+})+ $^2F_{7/2}$ (Yb^{3+}) → $^4I_{13/2}$ (Er^{3+})+ $^2F_{5/2}$ (Yb^{3+}).

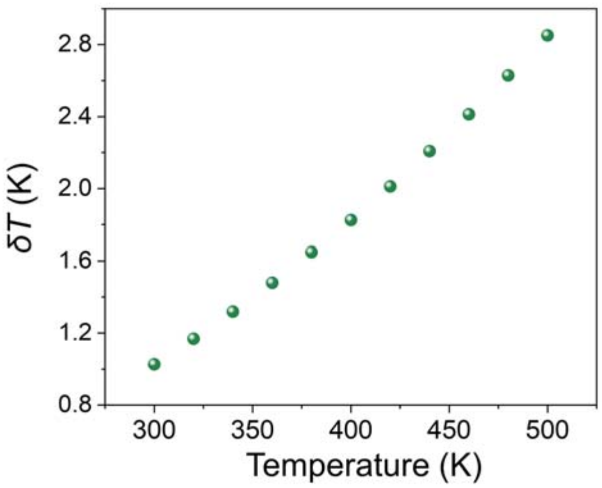

Figure S3. The temperature resolution of CSS:0.2Yb^{3+},0.02Er^{3+}.

Temperature resolution (δT) is an important parameter to characterize the performance of optical thermometer, which is defined as:[1–2]

$$\delta T = \frac{1}{S_r}\frac{\delta FIR}{FIR} \quad \text{(S1)}$$

Where $\delta FIR/FIR$ represents the relative standard deviation of the measurement. According to Equ. (1), the calculated temperature resolution δT is shown in Figure. S3 and the minimum value of δT is 1.03 K at 300 K.

References

1. Liu, S.; Cui, J.; Jia, J.; Fu, J.; You, W.; Zeng, Q.; Yang, Y.; Ye, X. High sensitive Ln^{3+}/Tm^{3+}/Yb^{3+} (Ln^{3+}=Ho^{3+}, Er^{3+}) tri-doped $Ba_3Y_4O_9$ upconverting optical thermometric materials based on diverse thermal response from non-thermally coupled energy levels. *Ceram. Int.* **2019**, *45*, 1–10.
2. Wu, H.; Hao, Z.; Zhang, L.; Zhang, X.; Xiao, Y.; Pan, G.-H.; Wu, H.; Luo, Y.; Zhang, L.; Zhang, J. Er^{3+}/Yb^{3+} codoped phosphor $Ba_3Y_4O_9$ with intense red upconversion emission and optical temperature sensing behavior. *J. Mater. Chem. C* **2018**, *6*, 3459–3467.
